# Enhancing Retention of an Internet-Based Cohort Study of Men Who Have Sex With Men (MSM) via Text Messaging: Randomized Controlled Trial 

**DOI:** 10.2196/jmir.2756

**Published:** 2013-08-27

**Authors:** Christine M Khosropour, Brent A Johnson, Alexandra V Ricca, Patrick S Sullivan

**Affiliations:** ^1^School of Public HealthDepartment of EpidemiologyUniversity of WashingtonSeattle, WAUnited States; ^2^Rollins School of Public HealthDepartment of EpidemiologyEmory UniversityAtlanta, GAUnited States; ^3^Rollins School of Public HealthDepartment of Biostatistics and BioinformaticsEmory UniversityAtlanta, GAUnited States

**Keywords:** HIV infections/prevention and control, prospective studies, Internet/organization and administration, SMS text messaging, homosexuality, male/statistics and numerical data

## Abstract

**Background:**

Black and Hispanic men who have sex with men (MSM) are disproportionately affected by HIV in the United States. The Internet is a promising vehicle for delivery of HIV prevention interventions to these men, but retention of MSM of color in longitudinal Internet-based studies has been problematic. Text message follow-up may enhance retention in these studies.

**Objective:**

To compare retention in a 12-month prospective Internet-based study of HIV-negative MSM randomized to receive bimonthly follow-up surveys either through an Internet browser online or through text messages.

**Methods:**

Internet-using MSM were recruited through banner advertisements on social networking and Internet-dating sites. White, black, and Hispanic men who were ≥18, completed an online baseline survey, and returned an at-home HIV test kit, which tested HIV negative, were eligible. Men were randomized to receive follow-up surveys every 2 months on the Internet or by text message for 12 months (unblinded). We used time-to-event methods to compare the rate of loss-to-follow-up (defined as non-response to a follow-up survey after multiple systematically-delivered contact attempts) in the 2 follow-up groups, overall and by race/ethnicity. Results are reported as hazard ratios (HR) and 95% confidence intervals (CI) of the rate of loss-to-follow-up for men randomized to text message follow-up compared to online follow-up.

**Results:**

Of 1489 eligible and consenting men who started the online baseline survey, 895 (60%) completed the survey and were sent an at-home HIV test kit. Of these, 710 of the 895 (79%) returned the at-home HIV test kit, tested HIV-negative, and were followed prospectively. The study cohort comprised 66% white men (470/710), 15% (106/710) black men, and 19% (134/710) Hispanic men. At 12 months, 77% (282/366) of men randomized to online follow-up were retained in the study, compared to 70% (241/344) men randomized to text message follow-up (HR=1.30, 95% CI 0.97-1.73). The rate of loss-to-follow-up was non-significantly higher in the text message arm compared to the online arm for both white (HR=1.43, 95% CI 0.97-1.73) and Hispanic men (HR=1.71, 95% CI 0.91-3.23); however, loss-to-follow-up among black men was non-significantly lower among those who received text message follow-up compared to online follow-up (HR=0.78, 95% CI 0.41-1.50). In the online arm, black men were significantly more likely to be lost to follow-up compared to white men (HR=2.25, 95% CI 1.36-3.71), but this was not the case in the text message arm (HR=1.23, 95% CI 0.70-2.16).

**Conclusions:**

We retained >70% of MSM enrolled in an online study for 12 months; thus, engaging men in online studies for a sufficient time to assess sustained outcomes is possible. Text message follow-up of an online cohort of MSM is feasible, and may result in higher retention among black MSM.

## Introduction

Over 60% of all new HIV diagnoses in the United States are among men who have sex with men (MSM) [1], who represent only an estimated 3%-7% of the US population [2]. From 2006-2009, HIV incidence increased by 34% among all young MSM aged 13-29, with an increased incidence of 48% noted among young black MSM [3]. Nearly three-quarters of the new HIV infections among young Hispanic Americans in 2009 were among MSM [3].

The recent increases in incidence among young MSM of color have led to a call for new approaches to HIV prevention [4], including technology-based prevention interventions [5]. The Internet is an attractive vehicle for intervention delivery for many reasons, including minimal cost relative to interventions utilizing human resources, standardization of intervention content [6], inclusion of high-risk MSM who may not be reached by in-person sampling methods [7,8], and recruitment of the large number of MSM required to use HIV incidence as a study endpoint [9,10]. Moreover, a recent meta-analysis showed that computer-delivered interventions are similarly efficacious to traditional, human-delivered interventions [6].

Despite the benefits of Internet-based interventions, retention in online cohort studies of MSM has been problematic. In three online studies of MSM, 3-month retention was between 15%-54% [11-13], below the 70% required by the Centers for Disease Control and Prevention (CDC) Prevention Research Synthesis criteria for best-evidence HIV prevention interventions [14]. Further, retention of black MSM in a number of online studies has been significantly lower than that of white MSM [12,13,15]; thus, results from these studies may not adequately represent those of black MSM or may accrue biases.

Differences in retention in online studies by race/ethnicity might be partially explained by differences in Internet access. In 2011, approximately 66% of white Americans had broadband Internet access in the household, compared to 49% of black and 51% of Hispanic Americans [16]. In contrast, mobile phone ownership among black and Hispanic Americans is equivalent to that of white Americans. National surveys conducted in 2012-2013 [17,18] indicate that 93% of black and 88% of Hispanic Americans owned a mobile phone compared to 90% of white Americans. Among mobile phone owners, a similar proportion of black and Hispanic Americans reported using text messaging (80% and 85%, respectively) compared to white Americans (79%), and 97% of young Americans aged 18-29 reported using their phones for SMS text messaging.

Because young black and Hispanic Americans are high users of mobile technology, we sought to investigate whether the use of text messaging would increase retention in a 12-month online cohort study of HIV-negative white, black, and Hispanic MSM. Our primary aims were to compare the 12-month retention of MSM randomized to receive online follow-up surveys versus text message follow-up surveys and to compare 12-month retention by race/ethnicity. We hypothesized that providing follow-up surveys by text messaging would result in higher retention, especially among MSM of color. Additionally, we describe mobile access to online surveys and the frequency of changing mobile phone numbers.

## Methods

### Study Design and Population

MSM were recruited from August to December 2010 by banner advertisements placed on social networking and select Internet-dating websites, including Facebook, Myspace, Black Gay Chat, and Adam4Adam. Website selection was based on data from four focus groups of MSM conducted in 2010. We chose websites that men indicated that they visited frequently or sites on which they felt the advertisements were “legitimate” and “trustworthy”. Eligible participants were male, at least 18 years of age, white non-Hispanic, black non-Hispanic, or Hispanic, and reported sex with a man in the past 12 months. Additional eligibility criteria included owning a mobile phone capable of sending and receiving text messages, being willing to receive an at-home HIV test kit, and not moving outside the United States in the next 12 months. Because we were interested in determining the retention of an Internet-based sample of HIV-negative MSM, only those men who returned their HIV test kit and tested HIV-negative were followed prospectively for 12 months.

Men provided electronic informed consent prior to initiating any study procedures by checking a box on the survey screen. Consenting men were asked to register for the study by providing an email address and mobile phone number before completing the baseline survey. These were validated in two sequential steps. First, a unique participant-specific URL for the baseline survey was sent to the participant’s email address. Second, participants who successfully linked into the baseline survey through the URL in their email were asked to enter their mobile phone number, to which a 3-digit code was sent by text message. Participants entered the 3-digit code on a survey screen in order to proceed in the study.

Men with verified email and mobile phone information completed a 60-minute baseline survey that included questions on condom acquisition and use, demographics, sexual risk behaviors, sexual partner history, and HIV testing history. At the conclusion of the baseline survey, men who did not report being HIV-positive provided their mailing address for an at-home HIV test kit. Those who provided a valid mailing address were randomized 1:1 to receive either text message or online follow-up surveys every 2 months for a total of 12 months. Randomization was implemented through the online enrollment system; there were no blocks of randomization, so men were assigned to an arm through random number generation at the time each man was determined to be eligible. Participants were not blinded to the arm to which they were randomized. To facilitate completion of the follow-up surveys, we asked participants to choose a preferred day of the week and time of day to receive their follow-up surveys. Additionally, we requested that participants indicate a preferred alternate contact method, in the event that we were unable to contact them via email (for the online arm) or text message (for the text message arm).

Participants were compensated US $15 for completing the baseline survey, US $10 for each follow-up survey, and US $15 for the Month 12 survey. Men were also paid US $20 for returning their at-home test kit. Payments were delivered via PayPal or Amazon.com electronic gift card after completion of each survey. Participants randomized to the text message arm who did not have a text message plan from their mobile phone carrier were charged US $0.10 per text message response. The cost for text messages sent to participants was paid by the research team. Before providing informed consent, potential participants were informed about the potential to incur costs associated with sending text messages as part of the study.

### Follow-Up

Participants received notifications to take their follow-up surveys 8 weeks after their last completed survey. Participants randomized to receive online follow-up surveys received an email that contained a unique URL to link to the follow-up survey. Participants randomized to receive text message follow-up surveys received a text message that provided an opportunity for participants to initiate the survey immediately or delay the survey for 24 hours. The text message survey was a question-and-response format (ie, the subsequent survey question was only sent once the response to the previous question had been received). Similar to the online survey, the text message survey incorporated skip patterns based on participant responses so that only relevant questions were asked. The content of the follow-up surveys, which queried men on their 2-month sexual history and HIV testing history, was identical for both randomization arms. Regardless of randomization arm, all participants received an email notification for the final (Month 12) survey, which was administered online.

We used a systematic arm-dependent method to maximize retention. Men randomized to the online arm who had not completed the survey 3 days after the initial notification email were automatically sent a reminder email. Two subsequent automated reminder emails were then sent, each separated by 24 hours. Men randomized to the text message arm who did not initially complete the survey or did not request a delay of survey initiation received 3 additional text message reminders, each separated by 24 hours. Men in both randomization arms who did not complete a follow-up survey after the first group of reminders were contacted up to 3 additional times by study staff, using the preferred method of contact provided in the baseline survey. As a final step, study staff called the participant via mobile phone to remind him to complete his follow-up survey. Participants were withdrawn from the study if they did not complete the follow-up survey after 3 phone calls.

### Outcome

The primary outcome was loss to follow-up, defined as administrative withdrawal by study staff (for non-response, as described above), before the Month 12 survey, or request by a participant to be withdrawn from the study.

### Statistical Analysis

Using methods for time-to-event data [19,20], we defined the period of analysis as the date of randomization until (1) the earliest of 365 days post-randomization or the date of completion of the Month 12 survey (for participants who were retained in the study); or (2) the date of the most recently completed survey (for participants who were lost to follow-up). Consequently, participants who were retained in the study but had not completed the final survey at the end of the analysis period (ie, 365 days after randomization) were considered censored.

Descriptive statistics were used to assess the distribution of participant characteristics by randomization arm, stratified by race/ethnicity. We used the Kaplan-Meier estimator to examine the rate of loss-to-follow-up by randomization arm and by race/ethnicity. We used Cox proportional hazards regression to estimate the hazard ratio (HR) and 95% confidence interval (CI) of time to loss-to-follow-up associated with randomization arm, overall, and stratified by race/ethnicity. We also estimated the HR and corresponding 95% CI of the rate of loss-to-follow-up within randomization arm for black and Hispanic participants relative to white participants.

We used scaled Schoenfeld residuals to evaluate the proportional hazards assumption of the Cox regression models [21]. For the primary model comparing randomization arms, a formal statistical test rejected the hypothesis of proportional hazards (*P*<.001). We determined that the relative hazard ratio changed sign at about 300 days (see Multimedia Appendix 1), which is consistent with the final time that men who were randomized to text message follow-up completed a text message survey. Therefore, we report the Cox regression estimates comparing online and text message follow-up for two models: one based on all data through Month 12 (365 days) and one based on data up to and including 300 days (Month 10). We did not detect a departure from the proportional hazards assumption for the model comparing retention by racial/ethnic group (*P*=.59); thus, we only report results using all data through Month 12 for that analysis. Reported *P*-values for all analyses are based on the Wald test of significance (alpha=0.05 level). Analyses were conducted in R and Stata 12.1.

All study procedures and analysis were reviewed and approved by the Institutional Review Board (IRB) of Emory University. This study used a randomized method for follow-up but did not meet the qualifications for ClinicalTrials.gov registration (ie, the study did not “prospectively assign human participants or groups of humans to one or more health-related interventions to evaluate the effects on health outcomes”.) This study did not utilize a health-related intervention or a health outcome.

## Results

### Study Population

**Figure 1:**
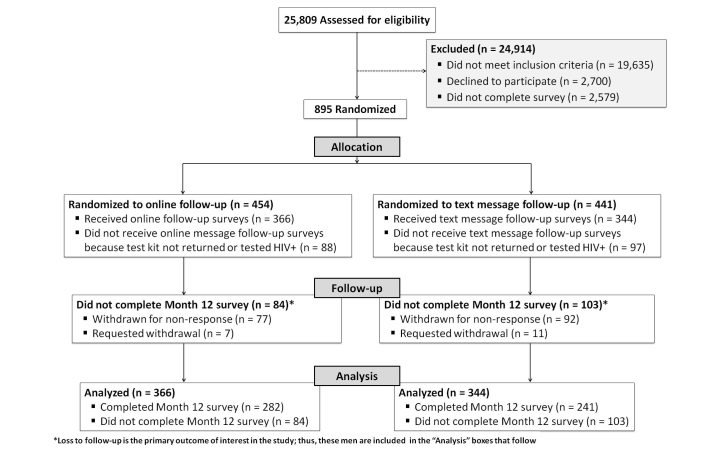
Enrollment of study participants in a 12-month prospective online study.

There were 6174 MSM eligible to participate in the study (Figure 1). Less than half of all consenting MSM (1489/3474) provided valid contact information and initiated the baseline survey. Of those who completed the baseline survey and were randomized (n=895), return of the at-home test kit was similar by randomization arm (81% online vs 78% text message, *P*=.34). In total, 710 MSM tested HIV-negative and were sent bimonthly follow-up surveys for 12 months.

Of 710 participants, 366 (52%) were randomized to online follow-up and 344 (49%) were randomized to text message follow-up. Two-thirds of participants were white and slightly over one-third were ≤24 years old (Table 1). Most men had at least some college-level education, and two-thirds resided in an urban area. Characteristics of participants between randomization arm did not differ within racial/ethnic groups. Characteristics were also balanced among men initially randomized to follow-up (n=895).

### Retention by Follow-Up Arm and Racial/Ethnic Group

Overall, 74% of men (523/710) were retained in the study at 12 months. Of the 187 men who were lost to follow-up, 18 (10%) requested to be withdrawn from the study and 169 (90%) were administratively withdrawn due to non-response. Withdrawal requests were more common among white men than black and Hispanic men: 13% (14/108) of white men, 8% (3/38) of black men, and 2% (1/41) of Hispanic men (*P=*.14) who were lost to follow-up requested to be withdrawn from the study (data not shown).

**Figure 2:**
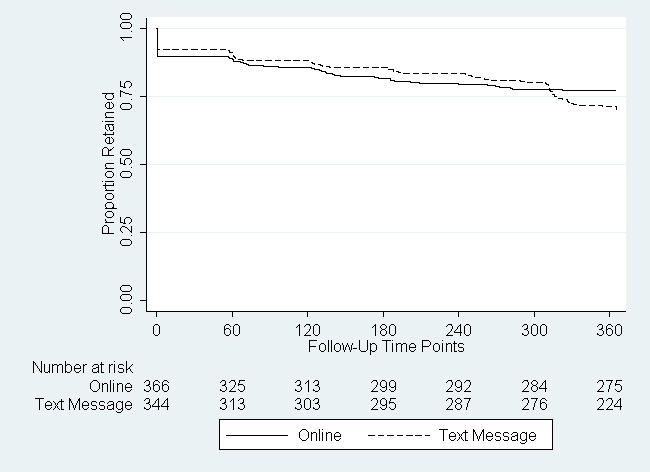
Retention of participants in an online study, by randomization arm (N=710).

Nearly 10% (65/710) of men were lost to follow-up before the first follow-up survey (Figure 2). At 12 months, men randomized to text message follow-up had a higher rate of loss-to-follow-up compared to men randomized to online follow-up, although this difference was not statistically significant (Table 2). Among white and Hispanic men, being randomized to text message follow-up was associated with a 42% and 71% higher rate of loss-to-follow-up, respectively, compared to online follow-up. In contrast, black men randomized to text message follow-up had a 20% reduction in the rate of loss-to-follow-up compared to black men randomized to online follow-up. Results using data only through Month 10 (300 days) did not differ (Table 2).

**Figure 3:**
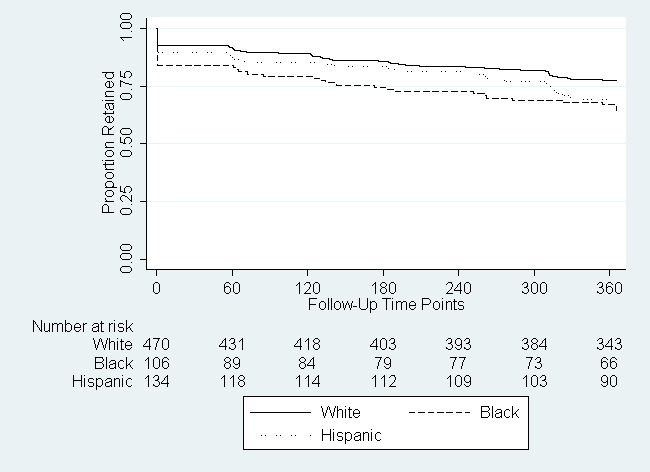
Retention of participants in an online study, by race/ethnicity (N=710).

Black men were less likely to be retained in the study compared to white or Hispanic men (Figure 3). Among men randomized to online follow-up, black men had a two-fold higher rate of loss-to-follow-up compared to white men (Table 3). However, there was no significant difference in the rate of loss-to-follow-up between black and white men randomized to text message follow-up. Compared to white men, Hispanic men randomized to text message follow-up had a somewhat higher rate of loss-to-follow-up.

Approximately 20% (71/362) of white men, 22% (15/68) of black men, and 17% (16/93) of Hispanic men who completed the Month 12 online survey did so on a mobile browser. Additionally, in a 5-month period beginning in February 2011, 27 of 244 (8%) participants in the text message arm notified study staff that they had acquired new mobile phone numbers. This included 6% (14/229) of white participants, 11% (5/46) of black participants, and 12% (8/69) of Hispanic participants.

**Table 1:** Baseline characteristics of participants in an online study, by race/ethnicity and randomization arm (N=710).^a, b^

			White		Black		Hispanic	
Characteristic		Total (N=710), n (%)	Online (N=241), n (%)	SMS (N=229), n (%)	Online (N=60), n (%)	SMS (N=46), n (%)	Online (N=65), n (%)	SMS (N=69), n (%)
**Age group**								
	18-24	263 (37.0)	71 (29.5)	79 (34.5)	28 (46.7)	25 (54.4)	28 (43.1)	32 (46.4)
	25-34	262 (36.9)	94 (39.0)	81 (35.4)	20 (33.3)	16 (34.8)	27 (41.5)	24 (34.8)
	35-44	107 (15.1)	43 (17.8)	39 (17.0)	7 (11.7)	4 (8.7)	6 (9.2)	8 (11.6)
	45-54	78 (11.0)	33 (13.7)	30 (13.1)	5 (8.3)	1 (2.2)	4 (6.2)	5 (7.3)
**Education**								
	≤ High school	130 (18.3)	41 (17.0)	42 (18.3)	14 (23.3)	5 (10.9)	10 (15.4)	18 (26.1)
	> High school / GED	580 (82.7)	200 (83.0)	187 (81.7)	46 (76.7)	41 (89.1)	55 (84.6)	51 (73.9)
**Geographic region** ^c^								
	West	197 (27.7)	56 (23.2)	63 (27.5)	9 (15.0)	4 (8.7)	25 (38.5)	40 (58.0)
	Midwest	96 (13.5)	41 (17.0)	32 (14.0)	4 (6.7)	6 (13.0)	6 (9.2)	7 (10.1)
	South	278 (39.1)	87 (36.0)	90 (39.3)	40 (66.7)	26 (56.5)	19 (29.2)	16 (23.2)
	Northeast	139 (19.6)	57 (23.7)	44 (19.2)	7 (11.7)	10 (21.7)	15 (23.1)	6 (8.7)
**Residence** ^d^								
	Urban^e^	459 (66.6)	150 (63.0)	141 (63.2)	47 (81.0)	31 (73.8)	46 (74.2)	44 (66.7)
	Rural	230 (33.4)	88 (37.0)	82 (36.8)	11 (19.0)	11 (26.2)	16 (25.8)	22 (33.3)
**Sexual identity**								
	Homosexual	603 (84.9)	218 (90.5)	201 (87.8)	40 (66.7)	31 (67.4)	53 (81.5)	60 (87.0)
	Bisexual	88 (12.4)	20 (8.3)	23 (10.0)	16 (26.7)	9 (19.6)	12 (18.5)	8 (11.6)
	Other	19 (2.7)	3 (1.2)	5 (2.2)	4 (6.7)	6 (13.0)	0 (0.0)	1 (1.5)
Ever tested for HIV		556 (78.5)	195 (81.3)	178 (78.1)	45 (75.0)	39 (84.8)	52 (80.0)	47 (68.1)
HIV test in past 12m		337 (47.6)	118 (49.2)	104 (45.6)	26 (43.3)	24 (52.2)	35 (53.9)	30 (43.5)
**Sex of SP, past 12m**								
	Men	660 (93.0)	229 (95.0)	216 (94.3)	55 (91.7)	36 (78.3)	60 (92.3)	64 (92.8)
	Men and women	50 (7.0)	12 (5.0)	13 (5.7)	5 (8.3)	10 (21.7)	5 (7.7)	5 (7.3)
**No. of MSP past 12m**								
	1	102 (14.5)	34 (14.1)	33 (14.4)	5 (8.6)	7 (15.6)	9 (14.1)	14 (20.3)
	2-5	300 (42.5)	99 (41.1)	103 (45.0)	27 (46.6)	19 (42.2)	25 (39.1)	27 (39.1)
	>5	304 (43.1)	108 (44.8)	93 (40.6)	26 (44.8)	19 (42.2)	30 (46.9)	28 (40.6)
UAI with MSP, past 12m		556 (83.0)	196 (86.0)	181 (82.7)	42 (73.7)	33 (82.5)	50 (80.7)	54 (84.4)

^a^Owing to missing data, numbers may not sum to column total. Denominators for proportions include those without missing data for that characteristic.

^b^Abbreviations—GED: general equivalency diploma; 12m: 12 months; (M)SP: (male) sex partner; UAI: unprotected anal intercourse.

^c^As defined by the US Census Bureau.

^d^Based on zip code where participant requested that at-home HIV test kit was sent.

^e^Urban defined as residence in a zip code with population ≥1000 per square mile.

**Table 2:** Rate of loss-to-follow-up among men participating in a 12-month online study randomized to text message versus online follow-up, overall and stratified by race/ethnicity (N=710).

		Proportion retained at 12 months n (%)^a^	Month 12 estimates		Month 10 estimates	
			Hazard ratio	95% CI	Hazard ratio	95% CI
**Overall**						
	Online	282/366 (77.1)	Referent	—	Referent	—
	Text message	241/344 (70.1)	1.30	0.97-1.73	1.28	0.96-1.71
**White**						
	Online	195/241 (80.9)	Referent	—	Referent	—
	Text message	167/229 (72.9)	1.43	0.97-2.09	1.41	0.96-2.06
**Black**						
	Online	37/60 (61.7)	Referent	—	Referent	—
	Text message	31/46 (67.4)	0.78	0.41-1.50	0.76	0.40-1.46
**Hispanic**						
	Online	50/65 (76.9)	Referent	—	Referent	—
	Text message	43/69 (62.3)	1.71	0.91-3.23	1.67	0.89-3.16

^a^Number retained out of total number defined by row.

**Table 3:** Rate of loss-to-follow-up among men participating in a 12-month online study randomized to text message versus online follow-up, by race/ethnicity and stratified by randomization arm (N=710).

		Proportion retained at 12 months, n (%)^a^	Hazard ratio	95% CI
**Overall**				
	White	362/470 (77.0)	Referent	—
	Black	68/106 (64.0)	1.70	1.17-2.45
	Hispanic	93/134 (69.4)	1.37	0.96-1.97
**Online follow-up**				
	White	195/241 (80.9)	Referent	—
	Black	37/60 (61.7)	2.25	1.36-3.71
	Hispanic	50/65 (76.9)	1.22	0.68-2.19
**Text message follow-up**				
	White	167/229 (72.9)	Referent	—
	Black	31/46 (67.4)	1.23	0.70-2.16
	Hispanic	43/69 (62.3)	1.47	0.94-2.35

^a^Number retained out of total number defined by row

## Discussion

### Principal Findings

In this large Internet-based cohort of MSM, nearly three-quarters of men were retained in the study for 12 months. Black men randomized to text message follow-up were somewhat more likely to be retained than those randomized to online follow-up, but this was not the case for white or Hispanic men. We observed a similar rate of loss-to-follow-up among black and white men randomized to text message follow-up, but black men followed exclusively online were less likely to be retained than white men. Among all men, about 1 in 5 completed an online survey via mobile phone browser.

Our demonstrated retention of 74% at 12 months is encouraging to investigators wishing to establish large Internet-based cohorts of MSM. This proportion retained is over three times that observed by our group in a 3-month prospective of MSM in 2009 [12] and greater than that reported from other online studies of MSM using 2-month [15], 3-month [11,13], or 6-month [22] study endpoints. Our retention is somewhat lower than that observed in a 2007 Internet-based study by Horvath et al [23], in which 85% of MSM were retained. Although our study and that of Horvath et al were similar in their approach and design, differences in the recruitment strategy between that study (ie, website banner advertisements and email contact to men who had previously participated in a study) and our study (ie, website banner advertisement only) may explain the lower retention among our study participants. Notably, in our study and the study of Horvath et al, loss-to-follow-up occurred most often in the period immediately following baseline and stabilized somewhat thereafter.

The high retention in our study can be attributed to a number of factors. First, we validated contact information from study participants, thereby excluding men who would have been lost to follow-up due to erroneous email addresses or mobile phone numbers. Second, based on results from our previous study which identified factors associated with retention in an online cohort of MSM [12], we encouraged participants to provide an email address that they checked daily. Third, we allowed participants to choose the day of week and time of day that they would like to be contacted to complete the follow-up surveys, which may have increased the convenience of survey completion. Fourth, we used a systematic follow-up method to encourage participants to complete their follow-up surveys. As part of our reminder protocol, we collected alternate contact information for all study participants so that changes in email address or mobile phone numbers would not result in a loss of contact with the participant. Finally, our study cohort included men who had completed the baseline survey and returned an at-home HIV test kit. Therefore, we selected for study participants who were actively engaged in the research study and more likely to be retained for the duration of the study.

Given the demonstrated need for new HIV prevention interventions among black MSM, it is promising that nearly two-thirds of black study participants were retained in this study. Although our 12-month retention of black men in this study is lower than the 3-month retention (78%) observed by Hightow-Weidmen et al at 3 months [24], the retention we observed is considerably higher than the comparable proportion in other prospective online studies of black MSM recruited exclusively online [12,13] and is close to the 70% set by the CDC’s criteria for best-evidence HIV prevention interventions [14]. The fact that black men randomized to text message follow-up had a higher retention than those followed exclusively online argues for the use of mobile measurement technologies to enhance research engagement in this group. Indeed, we noted a similar retention among black and white men in the text message arm, despite the relatively high loss-to-follow-up among black versus white men in the online follow-up. This latter observation is consistent with previous studies of MSM followed exclusively online [9,12,13,15] and highlights the challenges in equalizing retention by race/ethnicity in Internet-based settings where participants are not provided mobile-based options for data collection.

We were surprised that Hispanic men randomized to text message follow-up did not demonstrate a higher retention than those randomized to online follow-up, considering the high mobile phone usage by Hispanic Americans and relatively low household Internet access [16,18]. We speculate that the low retention in the text message arm may be partially explained by switching mobile phone numbers; 12% of Hispanic study participants contacted study staff within a 5-month period to inform us of a mobile phone number change, but we received no such notifications for email address changes. However, the proportion of changed mobile phone numbers was similar for Hispanic and black men; therefore, this does not completely explain the low retention we observed among Hispanic men who received text messages.

We noted that one-fifth of participants completed the Month 12 survey on their mobile phone. If the proportion of bimonthly online follow-up surveys completed via mobile Web is similar to that of the Month 12 survey, the higher retention in the online arm may be partially explained by mobile access to the survey. There are two important implications of this finding. First, research studies wishing to use Internet-based data collection may benefit from employing mobile-enabled Internet surveys. Second, studies should consider offering multiple methods of data collection. It is possible that men who completed surveys via mobile Web did so because they did not have household Internet access or because they preferred the convenience of a mobile phone. Either way, retention in studies may be enhanced by allowing participants to choose their preferred method of technology.

### Strengths and Limitations

This study has a number of strengths. We enrolled a large geographically and ethnically diverse cohort of MSM recruited from multiple websites. We employed a novel text message data collection system that incorporated skip patterns and recognition of invalid data entries. We used an automated reminder survey system that delivered surveys at specific times requested by study participants and sent automatic reminders at set time intervals.

There are several limitations to this analysis. First, we defined our study sample based on an event (return of the at-home HIV test kit) that occurred after randomization. Therefore, we may have lost some of the benefit of randomization to balance arms on confounding factors. Although characteristics of our study population were relatively similar by arm within racial/ethnic group, we cannot assess the distribution of unmeasured confounders. Second, our final study population included men who completed the baseline survey, provided valid contact information, and returned an at-home HIV test kit. Therefore, our population likely represents an actively engaged sample of research participants for which retention may be optimized. Third, although we specifically targeted websites to enhance recruitment of minority MSM (eg, Black Gay Chat), enrollment of black and Hispanic men was below that of white men. This was disappointing, given that the goal of this study was to assess retention in an Internet-based cohort of minority men. However, this was not unanticipated, as we have previously characterized the under-enrollment of black and Hispanic MSM in online research [9]. Probability-based sampling has the potential to address the second and third limitations, but validly implementing a rigorous, probability-based sampling scheme over the Internet is challenging. Fourth, data on usage of a mobile phone browser were systematically collected only for the Month 12 survey. Therefore, the extent to which men accessed the online survey on their mobile phone for the bimonthly surveys is unknown. Fifth, men in this study are not representative of MSM who do not use social networking or Internet dating sites, or who do not click on advertisements displayed on these sites. Finally, our auxiliary statistical analysis of retention rates (Multimedia Appendix 1) suggested that a time-varying coefficient Cox model (ie, one that allows the relative hazard ratio to fluctuate over time) may be more appropriate in future online studies. We addressed this potential limitation in the current analysis by analyzing and presenting all data as well as the subset of data that satisfied the proportional hazards assumption.

### Conclusions

In summary, we demonstrated an ability to retain >70% of MSM enrolled in an online study for 12 months. Our study suggests that follow-up via text message is feasible and may result in higher retention among black MSM. Based on our findings, it is possible to engage MSM at greatest risk for HIV infection in large prospective, Internet-based HIV prevention intervention studies using a time interval that is sufficient to assess sustained outcomes [25].

## Multimedia Appendix 1

Coefficient estimate for randomization arm effect: online versus text message.

## Multimedia Appendix 2

CONSORT-EHEALTH checklist V1.6.2 [25].

## Abbreviations

CDC: Centers for Disease Control and Prevention
IRB: Institutional Review Board
MSM: men who have sex with men
