# Supplementary material for: Enhancing Retention of an Internet-Based Cohort Study of Men Who Have Sex With Men (MSM) via Text Messaging: Randomized Controlled Trial
Source: J Med Internet Res. 2013 Aug 27;15(8):e194. doi: 10.2196/jmir.2756 (PMC3757960; doi:10.2196/jmir.2756)
Supplement: Supplementary file 1 [file jmir_v15i8e194_app1.pdf]

MULTIMEDIA APPENDIX 1

The plot of Schoenfeld residuals for the Cox proportional hazards regression comparing randomization arms is provided below. The open circles are weighted residuals while the solid line is the estimated log hazard ratio. The dashed line is an approximate point wise 95% confidence interval for the estimated nonlinear curve. If the estimated log hazard ratio is approximately linear throughout the follow-up period, then we would conclude that the ordinary proportional hazards assumption is adequate. Here, we detect a significant departure from non-linearity ($P<.001$) and determine that the relative log hazard changes signs at approximately 300 days.

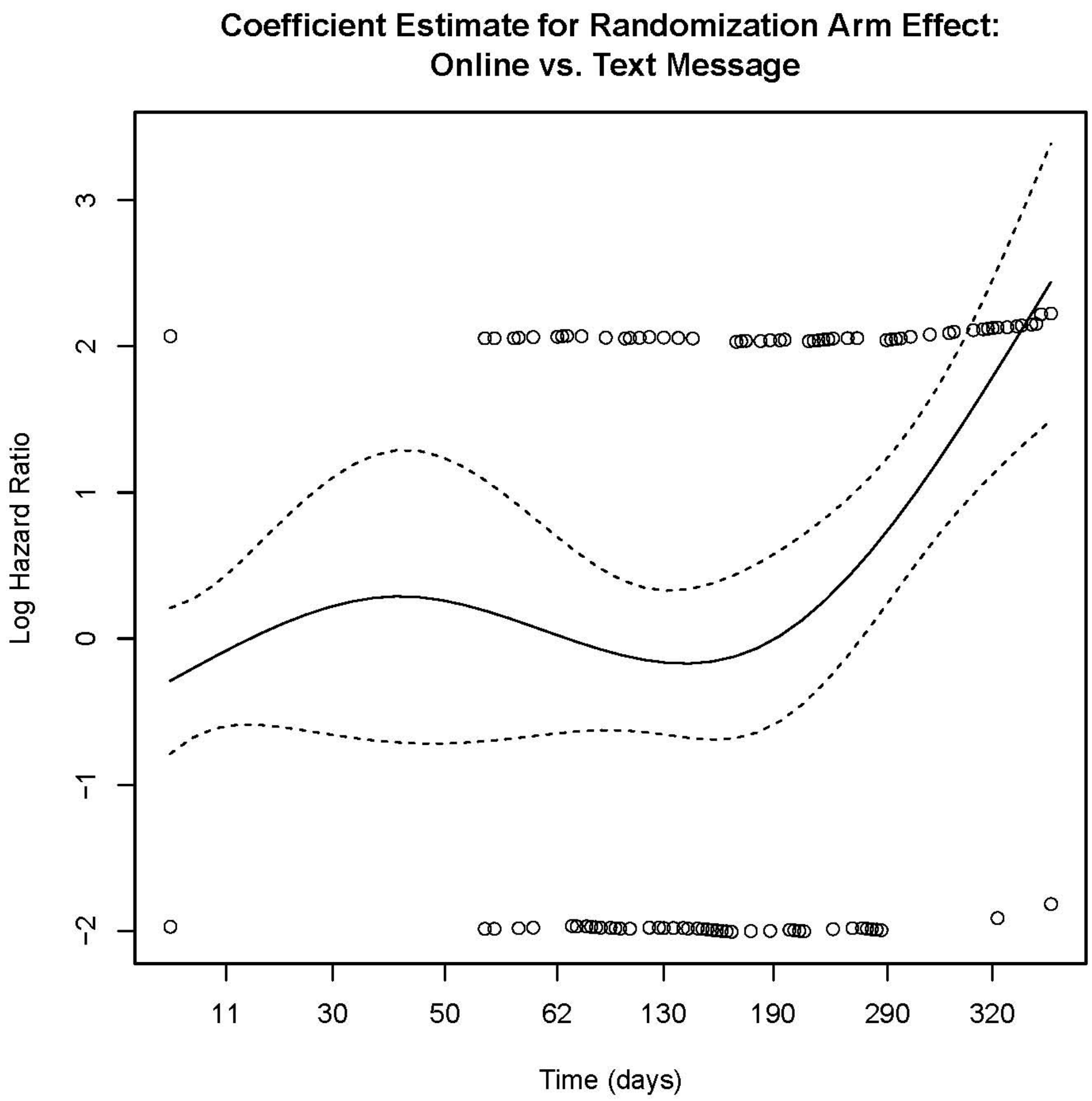
Coefficient Estimate for Randomization Arm Effect:
Online vs. Text Message
Log Hazard Ratio
3
2
1
0
−1
−2
11
30
50
62
130
190
290
320
Time (days)
