# Supplementary material for: Enhancing Retention of an Internet-Based Cohort Study of Men Who Have Sex With Men (MSM) via Text Messaging: Randomized Controlled Trial
Source: J Med Internet Res. 2013 Aug 27;15(8):e194. doi: 10.2196/jmir.2756 (PMC3757960; doi:10.2196/jmir.2756)
Supplement: Supplementary file 2 [file jmir_v15i8e194_app2.pdf]

CONSORT-EHEALTH Checklist V1.6.2 Report

(based on CONSORT-EHEALTH V1.6), available at [http://tinyurl.com/consort-ehealth-v1-6].

2756

Date completed

7/2/2013 17:58:57

by

Christine Khosropour

A randomized trial of text messaging to enhance retention in an Internet-based cohort study of men who have sex with men (MSM)

TITLE

1a-i) Identify the mode of delivery in the title

The title indicates that the study is a "randomized trial of text messaging to enhance retention in an Internet-based cohort study". Thus, the title identifies that the study is Internet-based and that text messaging was also used as a delivery mode.

1a-ii) Non-web-based components or important co-interventions in title

Not applicable - there were no non-web-based co-interventions in this study.

1a-iii) Primary condition or target group in the title

Yes, the title indicates that the study is conducted among "men who have sex with men", the primary target group.

ABSTRACT

1b-i) Key features/functionalities/components of the intervention and comparator in the METHODS section of the ABSTRACT

Yes, the abstract contains the following sentence to describe the components of the intervention and the comparator: "Men were randomized to receive follow-up surveys every two months on the Internet or by text message for 12 months"

1b-ii) Level of human involvement in the METHODS section of the ABSTRACT

This information is provided in the main Methods section of the paper. Although the intervention was fully automated, research staff did provide personal reminders (via email, text message or phone) to complete a follow-up survey. We feel that this level of detail is not appropriate for the abstract but we have explained the level of human involvement in detail in the Methods section.

1b-iii) Open vs. closed, web-based (self-assessment) vs. face-to-face assessments in the METHODS section of the ABSTRACT

This is not a blinded study because participants knew if they received online follow-up surveys or text message surveys. This information is in the abstract: "Men were randomized to receive follow-up surveys every two months on the Internet or by text message for 12 months (unblinded)". This sentence also states that surveys were conducted via the Internet or by text message.

1b-iv) RESULTS section in abstract must contain use data

Yes, the results contain the required data: "At 12 months, 282 (77%) of 366 men randomized to online follow-up were retained in the study, compared to 241 (70%) of 344 men randomized to text message follow-up (HR=1.30; 95% CI=0.97-1.73)."

1b-v) CONCLUSIONS/DISCUSSION in abstract for negative trials

This information is provided in the conclusion: "We retained >70% of MSM enrolled in an online study for 12 months; thus, engaging men in online studies for a sufficient time to assess sustained outcomes is possible. Text message follow-up of an online cohort of MSM is feasible, and may result in higher retention among black MSM"

INTRODUCTION

2a-i) Problem and the type of system/solution

Yes, this information is provided. The "problem" is described in 2a-ii below and we state the following objective: "Because young black and Hispanic Americans are high users of mobile technology, we sought to investigate whether the use of text messaging would increase retention in a 12-month, online cohort study of HIV-negative white, black, and Hispanic MSM"

2a-ii) Scientific background, rationale: What is known about the (type of) system

Yes, this information is provided: "Despite the benefits of Internet-based interventions, retention in online cohort studies of MSM has been problematic. In three online studies of MSM, 3-month retention was between 15%-54%, below the 70% required by the Centers for Disease Control and Prevention (CDC) Prevention Research Synthesis criteria for best-evidence HIV prevention interventions. Further, retention of black MSM in a number of online studies has been significantly lower than that of white MSM; thus, results from these studies may not adequately represent those of black MSM or may accrue biases." To our knowledge, there have not been previously published studies that have used text messaging to enhance retention in online research among MSM; thus we do not provide additional information about the use of similar systems.

METHODS

3a) CONSORT: Description of trial design (such as parallel, factorial) including allocation ratio

Yes, we provide the following objectives: "Our primary aims were to compare the 12-month retention of MSM randomized to receive online follow-up surveys versus text message follow-up surveys, and to compare 12-month retention by race/ethnicity. We hypothesized that providing follow-up surveys by text messaging would result in higher retention, especially among MSM of color."

3b) CONSORT: Important changes to methods after trial commencement (such as eligibility criteria), with reasons

There were no changes to eligibility criteria made after trial commencement.

3b-i) Bug fixes, Downtimes, Content Changes

There were no major content changes or bugs after the start of the trial of which we are aware. We conducted months of pilot-testing and formative work before beginning the trial to ensure the content of surveys was appropriate and that the text message system worked properly.

4a) CONSORT: Eligibility criteria for participants

Yes, we provide the following information: "Eligible participants were male, at least 18 years of age, white non-Hispanic, black non-Hispanic or Hispanic and reported sex with a man in the past 12 months. Additional eligibility criteria included owning a mobile phone capable of sending and receiving text messages, being willing to receive an at-home HIV test kit, and not moving outside the US in the next 12 months. Because we were interested in determining the retention of an Internet-based sample of HIV-negative MSM, only those men who returned their HIV test kit and tested HIV-negative were followed prospectively for 12 months."

4a-i) Computer / Internet literacy

We did not test the computer literacy of patients prior to obtaining informed consent. However, all patients were recruited from banner advertisements on social media and Internet-dating websites; thus, the computer literacy of patients was assumed.

4a-ii) Open vs. closed, web-based vs. face-to-face assessments:

Yes, we provide the following information about recruitment: "MSM were recruited from August to December 2010 by banner advertisements placed on social networking and select Internet-dating websites, including Facebook, MySpace, Black Gay Chat, and Adam4Adam"; the surveys "Participants received notifications to take their follow-up surveys eight weeks after their last completed survey. Participants randomized to receive online follow-up surveys received an email that contained a unique URL to link into the follow-up survey. Participants randomized to receive text message follow-up surveys received a text message which provided an opportunity for participants to initiate the survey immediately or delay the survey for 24 hours. The text message survey was a question and response format (i.e., the subsequent survey question was only sent once a response to the previous question had been received)"; and the follow-up personal contacts: "We used a systematic arm-dependent method to maximize retention. Men randomized to the online arm who had not completed the survey three days after the initial notification email were automatically sent a reminder email. Two subsequent automated reminder emails were then sent, each separated by 24 hours. Men randomized to the text message arm who did not initially complete the survey or did not request a delay of survey initiation received three additional text message reminders, each separated by 24 hours. Men in both randomization arms who did not complete a follow-up survey after the first group of reminders were contacted up to three additional times by study staff, using the preferred method of contact provided in the baseline survey. As a final step, study staff called the participant via mobile phone to remind him to complete his follow-up survey. Participants were withdrawn from the study if they did not complete the follow-up survey after three phone calls."

4a-iii) Information giving during recruitment

All informed consent procedures conformed to requirements set by the Emory University Institutional Review Board; in the manuscript we state that: "All study procedures and analysis were reviewed and approved by the Institutional Review Board (IRB) of Emory University." We provide the following information: "Men provided electronic informed consent prior to initiating any study procedures"

4b) CONSORT: Settings and locations where the data were collected

We provide the following information regarding data collection: "Participants received notifications to take their follow-up surveys eight weeks after their last completed survey. Participants randomized to receive online follow-up surveys received an email that contained a unique URL to link into the follow-up survey. Participants randomized to receive text message follow-up surveys received a text message which provided an opportunity for participants to initiate the survey immediately or delay the survey for 24 hours. The text message survey was a question and response format (i.e., the subsequent survey question was only sent once a response to the previous question had been received). Similarly to the online survey, the text message survey incorporated skip patterns based on participant responses so that only relevant questions were asked. The content of the follow-up surveys, which queried men on their 2-month sexual history and HIV testing history, was identical for both randomization arms. Regardless of randomization arm, all participants received an email notification for the final (month 12) survey, which was administered online."

4b-i) Report if outcomes were (self-)assessed through online questionnaires

We provide information about the outcomes in this study: "The primary outcome was loss to follow-up, defined as administrative withdrawal by study staff (for non-response, as described above) before the month 12 survey, or request by a participant to be withdrawn from the study."

4b-ii) Report how institutional affiliations are displayed

We did not include this information because we did not feel it is relevant to this study.

5) CONSORT: Describe the interventions for each group with sufficient details to allow replication, including how and when they were actually administered

5-i) Mention names, credential, affiliations of the developers, sponsors, and owners

There was no intervention in this study. It was randomization to one of two methods of data collection; thus there is no information provided on the developers, sponsors, etc.

5-ii) Describe the history/development process

Per 5-i, there is no "intervention" for this study. Randomization was to one of two methods of data collection.

5-iii) Revisions and updating

Per 5-i, there is no "intervention" for this study. Randomization was to one of two methods of data collection.

5-iv) Quality assurance methods

The paper does not directly address this sub-item. The primary outcome of this paper was to measure 12-month retention, which was determined by completion of a survey; thus we did not use specific quality assurance methods in this paper.

5-v) Ensure replicability by publishing the source code, and/or providing screenshots/screen-capture video, and/or providing flowcharts of the algorithms used

Per 5-i, there is no "intervention" for this study, thus there is no specific algorithm to share. Randomization was to one of two methods of data collection.

5-vi) Digital preservation

There is no intervention for this study, thus there is no specific digital presentation of the application. Randomization was to one of two methods of data collection.

5-vii) Access

We provide the following information about the baseline survey: "Men with verified email and mobile phone information completed a 60-minute baseline survey that included questions on condom acquisition and use, demographics, sexual risk behaviors, sexual partner history, and HIV testing history. At the conclusion of the baseline survey, men who did not report being HIV-positive provided their mailing address for an at-home HIV test kit"; and the follow-up surveys: "Participants received notifications to take their follow-up surveys eight weeks after their last completed survey. Participants randomized to receive online follow-up surveys received an email that contained a unique URL to link into the follow-up survey. Participants randomized to receive text message follow-up surveys received a text message which provided an opportunity for participants to initiate the survey immediately or delay the survey for 24 hours. The text message survey was a question and response format (i.e., the subsequent survey question was only sent once a response to the previous question had been received)"

5-viii) Mode of delivery, features/functionalities/components of the intervention and comparator, and the theoretical framework

There is no "intervention" for this study; randomization was to one of two methods of data collection. We provide the following information about the two randomization arms: "Those who provided a valid mailing address were randomized 1:1 to receive either text message or online follow-up surveys every two months for a total of 12 months"; and "The content of the follow-up surveys, which queried men on their 2-month sexual history and HIV testing history, was identical for both randomization arms" regarding the content of the surveys.

5-ix) Describe use parameters

We provide the following information about timing of the follow-up surveys (i.e., the two arms for the study): "Those who provided a valid mailing address were randomized 1:1 to receive either text message or online follow-up surveys every two months for a total of 12 months."

5-x) Clarify the level of human involvement

We provide the following information to indicate that the follow-up surveys and reminders were automated, but that additional follow-up was conducted by research staff: "To facilitate completion of the follow-up surveys, we asked participants to choose a preferred day of the week and time of day to receive their follow-up surveys. Additionally, we requested that participants indicate a preferred alternate contact method, in the event that we were unable to contact them via email (for the online arm) or text message (for the text message arm)"; and "Participants received notifications to take their follow-up surveys eight weeks after their last completed survey. Participants randomized to receive online follow-up surveys received an email that contained a unique URL to link into the follow-up survey. Participants randomized to receive text message follow-up surveys received a text message which provided an opportunity for participants to initiate the survey immediately or delay the survey for 24 hours"; and "Men randomized to the online arm who had not completed the survey three days after the initial notification email were automatically sent a reminder email. Two subsequent automated reminder emails were then sent, each separated by 24 hours. Men randomized to the text message arm who did not initially complete the survey or did not request a delay of survey initiation received three additional text message reminders, each separated by 24 hours. Men in both randomization arms who did not complete a follow-up survey after the first group of reminders were contacted up to three additional times by study staff, using the preferred method of contact provided in the baseline survey. As a final step, study staff called the participant via mobile phone to remind him to complete his follow-up survey. Participants were withdrawn from the study if they did not complete the follow-up survey after three phone calls."

5-xi) Report any prompts/reminders used

We provide the following section regarding reminders: "We used a systematic arm-dependent method to maximize retention. Men randomized to the online arm who had not completed the survey three days after the initial notification email were automatically sent a reminder email. Two subsequent automated reminder emails were then sent, each separated by 24 hours. Men randomized to the text message arm who did not initially complete the survey or did not request a delay of survey initiation received three additional text message reminders, each separated by 24 hours. Men in both randomization arms who did not complete a follow-up survey after the first group of reminders were contacted up to three additional times by study staff, using the preferred method of contact provided in the baseline survey. As a final step, study staff called the participant via mobile phone to remind him to complete his follow-up survey. Participants were withdrawn from the study if they did not complete the follow-up survey after three phone calls."

5-xii) Describe any co-interventions (incl. training/support)

There were no co-interventions in this study.

6a) CONSORT: Completely defined pre-specified primary and secondary outcome measures, including how and when they were assessed

Yes, we provide the following information on the outcome: "The primary outcome was loss to follow-up, defined as administrative withdrawal by study staff (for non-response, as described above) before the month 12 survey, or request by a participant to be withdrawn from the study" and how it was assessed: "Using methods for time-to-event data, we defined the period of analysis as the date of randomization until: (1) the earliest of 365 days post-randomization or the date of completion of the month 12 survey (for participants who were retained in the study); or (2) the date of the most recently completed survey (for participants who were lost to follow-up). Consequently, participants who were retained in the study but had not completed the final survey at the end of the analysis period (i.e., 365 days after randomization) were considered censored....We used the Kaplan-Meier estimator to examine the rate of loss to follow-up by randomization arm and by race/ethnicity. We used Cox proportional hazards regression to estimate the hazard ratio (HR) and 95% confidence interval (CI) of time to loss to follow-up associated with randomization arm, overall and stratified by race/ethnicity. We also estimated the HR and corresponding 95% CI of the rate of loss to follow-up within randomization arm for black and Hispanic participants relative to white participants."

6a-i) Online questionnaires: describe if they were validated for online use and apply CHERRIES items to describe how the questionnaires were designed/deployed

The primary outcome was not obtained from a questionnaire; it was retention in the study. Therefore, we did not provide this information

6a-ii) Describe whether and how "use" (including intensity of use/dosage) was defined/measured/monitored

We provide the following description of use, in our case, retention in the study, as the following: "The primary outcome was loss to follow-up, defined as administrative withdrawal by study staff (for non-response, as described above) before the month 12 survey, or request by a participant to be withdrawn from the study."

6a-iii) Describe whether, how, and when qualitative feedback from participants was obtained

We do not provide information about qualitative feedback from participants since we did not have a true intervention; however that information will be included in a separate paper.

6b) CONSORT: Any changes to trial outcomes after the trial commenced, with reasons

We do not include information on changes to the trial because our protocol did not change after enrollment commenced.

7a) CONSORT: How sample size was determined

7a-i) Describe whether and how expected attrition was taken into account when calculating the sample size

Because our primary outcome was retention in the study (i.e., loss to follow-up), we did not calculate a sample size for the study.

7b) CONSORT: When applicable, explanation of any interim analyses and stopping guidelines

Not applicable.

8a) CONSORT: Method used to generate the random allocation sequence

We provide the following information: "Randomization was implemented through the online enrollment system; there were no blocks of randomization, so men were assigned to an arm through random number generation at the time each man was determined to be eligible."

8b) CONSORT: Type of randomisation; details of any restriction (such as blocking and block size)

We provide the following information: "Randomization was implemented through the online enrollment system; there were no blocks of randomization, so men were assigned to an arm through random number generation at the time each man was determined to be eligible."

9) CONSORT: Mechanism used to implement the random allocation sequence (such as sequentially numbered containers), describing any steps taken to conceal the sequence until interventions were assigned

We provide the following information: "Randomization was implemented through the online enrollment system; there were no blocks of randomization, so men were assigned to an arm through random number generation at the time each man was determined to be eligible."

10) CONSORT: Who generated the random allocation sequence, who enrolled participants, and who assigned participants to interventions

We provide the following information: "Randomization was implemented through the online enrollment system; there were no blocks of randomization, so men were assigned to an arm through random number generation at the time each man was determined to be eligible."

11a) CONSORT: Blinding - If done, who was blinded after assignment to interventions (for example, participants, care providers, those assessing outcomes) and how

11a-i) Specify who was blinded, and who wasn't

We provide the following information: "Participants were not blinded to the arm to which they were randomized."

11a-ii) Discuss e.g., whether participants knew which intervention was the "intervention of interest" and which one was the "comparator"

There is no intervention for this study, thus there is no intervention of interest. Randomization was to one of two methods of data collection.

11b) CONSORT: If relevant, description of the similarity of interventions

Not applicable.

12a) CONSORT: Statistical methods used to compare groups for primary and secondary outcomes

We provide the following information: "Using methods for time-to-event data, we defined the period of analysis as the date of randomization until: (1) the earliest of 365 days post-randomization or the date of completion of the month 12 survey (for participants who were retained in the study); or (2) the date of the most recently completed survey (for participants who were lost to follow-up). Consequently, participants who were retained in the study but had not completed the final survey at the end of the analysis period (i.e., 365 days after randomization) were considered censored.

Descriptive statistics were used to assess the distribution of participant characteristics by randomization arm, stratified by race/ethnicity. We used the Kaplan-Meier estimator to examine the rate of loss to follow-up by randomization arm and by race/ethnicity. We used Cox proportional hazards regression to estimate the hazard ratio (HR) and 95% confidence interval (CI) of time to loss to follow-up associated with randomization arm, overall and stratified by race/ethnicity. We also estimated the HR and corresponding 95% CI of the rate of loss to follow-up within randomization arm for black and Hispanic participants relative to white participants."

12a-i) Imputation techniques to deal with attrition / missing values

The primary outcome was retention/loss-to-follow-up; therefore, participants who did not complete the study were still included in the analysis and this data was not missing. We provide the following information about the primary outcome: "The primary outcome was loss to follow-up, defined as administrative withdrawal by study staff (for non-response, as described above) before the month 12 survey, or request by a participant to be withdrawn from the study."

12b) CONSORT: Methods for additional analyses, such as subgroup analyses and adjusted analyses

We did not included additional analyses.

RESULTS

13a) CONSORT: For each group, the numbers of participants who were randomly assigned, received intended treatment, and were analysed for the primary outcome

This information is provided in Figure 1.

13b) CONSORT: For each group, losses and exclusions after randomisation, together with reasons

This information is provided in Figure 1.

13b-i) Attrition diagram

This information is provided in Figure 1.

14a) CONSORT: Dates defining the periods of recruitment and follow-up

Yes, we provide the following information: "MSM were recruited from August to December 2010"; and " Those who provided a valid mailing address were randomized 1:1 to receive either text message or online follow-up surveys every two months for a total of 12 months"

14a-i) Indicate if critical “secular events” fell into the study period

This is not applicable to our study.

14b) CONSORT: Why the trial ended or was stopped (early)

The trial was not stopped early; thus this is not discussed in the paper.

15) CONSORT: A table showing baseline demographic and clinical characteristics for each group

Yes, this information is provided in Table 1 of the study.

15-i) Report demographics associated with digital divide issues

The required demographics are provided in Table 1.

16a) CONSORT: For each group, number of participants (denominator) included in each analysis and whether the analysis was by original assigned groups

16-i) Report multiple “denominators” and provide definitions

This information is provided Figure 1.

16-ii) Primary analysis should be intent-to-treat

Because the primary outcome of the study was to assess retention at 12 months, we, by definition, included those who were non-users. This information is provided in the objective "The primary outcome was loss to follow-up, defined as administrative withdrawal by study staff (for non-response, as described above) before the month 12 survey, or request by a participant to be withdrawn from the study." and Figure 1.

17a) CONSORT: For each primary and secondary outcome, results for each group, and the estimated effect size and its precision (such as 95% confidence interval)

The effect size and precision of each outcome is information is provided in Tables 2 and 3.

17a-i) Presentation of process outcomes such as metrics of use and intensity of use

This is not applicable to this study.

17b) CONSORT: For binary outcomes, presentation of both absolute and relative effect sizes is recommended

The proportions and relative effect sizes are presented in Tables 2 and 3.

18) CONSORT: Results of any other analyses performed, including subgroup analyses and adjusted analyses, distinguishing pre-specified from exploratory

Not applicable.

18-i) Subgroup analysis of comparing only users

The nature of this study was to compare retention in the study; thus by definition we were comparing users to non-users.

19) CONSORT: All important harms or unintended effects in each group

Our study is not a randomized controlled trial per ClinicalTrials.gov requirements; thus we do not include this information.

19-i) Include privacy breaches, technical problems

Our study is not a randomized controlled trial per ClinicalTrials.gov requirements; thus we do not include this information.

19-ii) Include qualitative feedback from participants or observations from staff/researchers

Feedback from participants is being analyzed and will be presented in a separate paper.

DISCUSSION

20) CONSORT: Trial limitations, addressing sources of potential bias, imprecision, multiplicity of analyses

20-i) Typical limitations in ehealth trials

We provide the following limitations: "First, we defined our study sample based on an event (return of the at-home HIV test kit) that occurred after randomization. Therefore, we may have lost some of the benefit of randomization to balance arms on confounding factors. Although characteristics of our study population were relatively similar by arm within racial/ethnic group, we cannot assess the distribution of unmeasured confounders. Second, our final study population included men who completed the baseline survey, provided valid contact information, and returned an at-home HIV test kit. Therefore our population likely represents an actively engaged sample of research participants for which retention may be optimized. Third, although we specifically targeted websites to enhance recruitment of minority MSM (e.g., Black Gay Chat), enrollment of black and Hispanic was below that of white men. This was disappointing, given that the goal of this study was to assess retention in an online cohort of minority men. However, this was not unanticipated, as we have previously characterized the under-enrollment of black and Hispanic MSM in online research. Fourth, data on mobile phone browser was only systematically collected for the Month 12 survey. Therefore, the extent to which men accessed the online survey on their mobile phone for earlier surveys is unknown. Fifth, men in this study are not representative of MSM who do not use social networking or Internet dating sites or who do not click on advertisements displayed on these sites. Finally, our auxiliary statistical analysis of retention rates (Multimedia Appendix) suggested that a time-varying coefficient Cox model, (i.e., one that allows the relative hazard ratio to fluctuate over time), may be more appropriate in future online studies. We addressed this potential limitation in the current analysis by analyzing and presenting all data as well as the subset of data that satisfied the proportional hazards assumption."

21) CONSORT: Generalisability (external validity, applicability) of the trial findings

21-i) Generalizability to other populations

We acknowledge that "men in this study are not representative of MSM who do not use social networking or Internet dating sites or who do not click on advertisements displayed on these sites"

21-ii) Discuss if there were elements in the RCT that would be different in a routine application setting

This is not discussed in this paper since we did not use a true intervention.

22) CONSORT: Interpretation consistent with results, balancing benefits and harms, and considering other relevant evidence

22-i) Restate study questions and summarize the answers suggested by the data, starting with primary outcomes and process outcomes (use)

We provide the following information in the first paragraph of the discussion: "Black men randomized to text message follow-up were somewhat more likely to be retained than those randomized to online follow-up, but this was not the case for white or Hispanic men. We observed a similar rate of loss to follow-up among black and white men randomized to text message follow-up, but black men followed exclusively online were less likely to be retained than white men."

22-ii) Highlight unanswered new questions, suggest future research

We provide the following suggestions for future research: "First, research studies wishing to use Internet-based data collection may benefit from employing mobile-enabled Internet surveys. Second, studies should consider offering multiple methods of data collection. It is possible that men who completed surveys via mobile web did so because they did not have household Internet access, or because they preferred the convenience of a mobile phone. Either way, retention in studies may be enhanced by allowing participants to choose their preferred method of technology."

Other information

23) CONSORT: Registration number and name of trial registry

This trial was not registered on ClinicalTrials.gov because it did not meet the requirements of registration. The current guidelines state that researchers must register: “any research study that prospectively assigns human participants or groups of humans to one or more health-related interventions to evaluate the effects on health outcomes.” The text message/online follow-up survey intervention was not “health-related” and our outcome, retention in the study at 12 months, was not a heath outcome. Therefore, this trial was not registered. We have included a sentence in the Methods section of the paper to explain why the trial was not registered.

24) CONSORT: Where the full trial protocol can be accessed, if available

The trial protocol is not available for open access since this trial was not registered.

25) CONSORT: Sources of funding and other support (such as supply of drugs), role of funders

Yes, we provide the following information: "The author(s) disclose receipt of the following financial support for the research and/or authorship of this article: National Center for Minority Health and Health Disparities (1RC1MD004370) and Emory Center for AIDS Research (P30 AI050409)."

X26-i) Comment on ethics committee approval

We provide the following information: "All study procedures and analysis were reviewed and approved by the Institutional Review Board (IRB) of Emory University."

x26-ii) Outline informed consent procedures

We provide the following information regarding informed consent: "Men provided electronic informed consent prior to initiating any study procedures by checking a box on the survey screen."

X26-iii) Safety and security procedures

Not applicable.

X27-i) State the relation of the study team towards the system being evaluated

We provide the following information: "The author(s) declare no conflicts of interest with respect to the authorship and/or publication of this article."
